# Influence of social and material individual and area deprivation on suicide mortality among 2.7 million Canadians: A prospective study

**DOI:** 10.1186/1471-2458-11-577

**Published:** 2011-07-19

**Authors:** Stephanie Burrows, Nathalie Auger, Philippe Gamache, Danielle St-Laurent, Denis Hamel

**Affiliations:** 1 Centre de recherche du Centre hospitalier de l'Université de Montréal, 1301 rue Sherbrooke Est Montréal, Québec, H2L 1M3, Canada; 2 Institut national de santé publique du Québec, Montréal, Canada; 3 Université du Québec à Montréal, Montréal, Canada; 4 Département Médecine Sociale et Préventive, Université de Montréal, Montréal, Canada

## Abstract

**Background:**

Few studies have investigated how area-level deprivation influences the relationship between individual disadvantage and suicide mortality. The aim of this study was to examine individual measures of material and social disadvantage in relation to suicide mortality in Canada and to determine whether these relationships were modified by area deprivation.

**Methods:**

Using the 1991-2001 Canadian Census Mortality Follow-up Study cohort (N = 2,685,400), measures of individual social (civil status, family structure, living alone) and material (education, income, employment) disadvantage were entered into Cox proportional hazard models to calculate hazard ratios (HR) and 95% confidence intervals (CI) for male and female suicide mortality. Two indices of area deprivation were computed - one capturing social, and the other material, dimensions - and models were run separately for high versus low deprivation.

**Results:**

After accounting for individual and area characteristics, individual social and material disadvantage were associated with higher suicide mortality, especially for individuals not employed, not married, with low education and low income. Associations between social and material area deprivation and suicide mortality largely disappeared upon adjustment for individual-level disadvantage. In stratified analyses, suicide risk was greater for low income females in socially deprived areas and males living alone in materially deprived areas, and there was no evidence of other modifying effects of area deprivation.

**Conclusions:**

Individual disadvantage was associated with suicide mortality, particularly for males. With some exceptions, there was little evidence that area deprivation modified the influence of individual disadvantage on suicide risk. Prevention strategies should primarily focus on individuals who are unemployed or out of the labour force, and have low education or income. Individuals with low income or who are living alone in deprived areas should also be targeted.

## Background

Suicide is shaped by social forces and, perhaps more than other common causes of death, reflects how individuals relate to society [1,2]. Many studies evaluate suicide from either a place-based or an individual-based perspective, but few incorporate both. Individual-level studies have examined suicide with respect to material (lack of goods, services, resources and amenities) and social (lack of social cohesion, co-operation, mutual assistance and trust) disadvantage [3]. The direction and strength of associations, however, varies by sex, measure of disadvantage, time period or region [4-6]. Some studies find no association between socioeconomic status and suicide [7-11], whereas others report higher risks with low education [12], income [13,14], occupational position [15], unemployment [13,14], or mixed results depending on the socioeconomic indicator [16] or sex [17]. Being single is associated with suicide in some studies [8,11,15] but not others [7,17]. Although divorced/separated individuals are usually at greater risk than their married counterparts, studies are conflicting with some reporting greater risks for men [17] and others for women [8].

Contextual environment is another important element, and several studies have documented associations between suicide and area-level material and social characteristics [5,6]. Area attributes may not only influence overall suicide rates in geographic regions, but their influence may also depend on residents' personal characteristics such as marital or socioeconomic status [13]. Research in the US [18] and Denmark [13] suggests that associations between individual disadvantage and suicide were not affected by adjustment for neighbourhood socioeconomic characteristics. Area characteristics may, however, have modifying effects on the association between individual factors and suicide. One possibility is that the relationship between individual disadvantage and suicide is stronger in deprived areas. Several mechanisms have been proposed to describe pathways through which this may occur [19]. Better-off individuals may avert harmful effects of deprived areas, through greater capacity to afford goods and services, thereby increasing resilience to environmental factors. It is also possible that worse-off individuals living in advantaged areas could reduce their risk by benefiting disproportionately from the better material and social infrastructure (e.g., public amenities, job opportunities) of these areas; whereas combined individual disadvantage and a deprived environment may accumulate and increase risk for those living in deprived areas.

Another possibility is that the relationship between individual disadvantage and suicide might be weaker in deprived areas. Durkheim's theory of social integration [2] and Gibbs and Martin's status integration theory [20,21] that propose that individuals derive mental, emotional and physical benefits when they interact, connect and validate each other within a community support this possibility. These processes might be greater in deprived areas where disadvantaged individuals may feel more connected with other residents who are more similar to them (lowering isolation, depression, and suicide risk) compared with advantaged areas where dissonance may be greater (increasing suicide risk). Alternatively, the relationship between individual disadvantage and suicide might be the same across advantaged and deprived areas, suggesting little influence of area characteristics on suicide risk.

To our knowledge, only two studies assessed if the effects of individual socioeconomic characteristics vary in socioeconomically different areas [13,19]. In both studies, there was little evidence that area deprivation modified the influence of individual disadvantage on suicide, and the results that did support interaction were inconsistent, with individual disadvantage a stronger determinant of suicide in deprived areas for some measures and a weaker determinant for others. For instance, in Finland the difference in alcohol-related suicide rates between manual and white-collar workers was larger in deprived areas than in advantaged ones, whereas the difference in non-alcohol-related suicides was smaller in deprived than in advantaged areas [19]. Depending on age and sex, unmarried and unemployed individuals in Denmark had a higher risk of suicide in deprived areas, but low income individuals had higher risk in advantaged areas [13].

There is a need to understand whether individual- and area-level deprivation interact to influence suicide in order to inform policies and interventions to reduce suicide. Such actions are essential in Canada where suicide is a leading cause of death, especially among middle-aged adults who recorded 12-22 deaths annually per 100,000 inhabitants from 1984-2004 [22,23]. To our knowledge, no Canadian study has used individual and area information simultaneously to investigate suicide. Given that the available evidence on effect modification is limited to two European studies with few measures of social and/or material deprivation, and that the results are inconclusive, we sought to evaluate if more definitive results could be reached in a different setting with a full spectrum of social and material deprivation indicators. The aim of this study was to examine the relationship between individual measures of material and social disadvantage and suicide in Canada and to determine whether relationships were modified by area deprivation.

## Methods

### Data

We extracted data from the 1991-2001 Canadian Census Mortality Follow-up Study cohort. Respondents to the long-form of the 1991 Census who resided in the 10 Canadian provinces were linked to Canadian mortality data over a follow-up of 10.6 years [24]. The cohort represents approximately 15% of the Canadian non-institutionalized population aged 25+ years at baseline (N = 2,735,152). Ethical approval for the follow-up study was obtained from the Statistics Canada Policy Committee and the research ethics committee of the University of Toronto.

### Variables

Suicides were identified using International Classification of Diseases (ICD) codes for principal cause of death (ICD-9 E950-E959; ICD-10 X60-84, Y87.0). "Undetermined" deaths (ICD-9 E980-E989; ICD-10 Y10-Y34, Y87.2, and Y89.9) were also included since they may represent misclassified suicide cases [25,26]. The tenth revision of the ICD was implemented in 2000.

Two composite indices capturing social and material dimensions of deprivation were calculated for Canadian census enumeration areas (EA), small areas containing 750 inhabitants on average [27], through a principal component analysis of six census variables [28-30]. The social deprivation index accounts for variations in the proportion of separated, divorced or widowed individuals, proportion of single-parent families, and proportion of persons living alone. The material deprivation index accounts for variations in the proportion of persons with no high school diploma, ratio of employment to population, and average income. For both indices, EAs were ranked as population-weighted quintiles. EAs with small population counts or containing mainly institutionalized residents (approximately 1% of the cohort, 1.3% of suicides), for which the index could not be calculated, were excluded [28]. The final sample contained 2,685,400 individuals.

Individual-level variables capturing social and material disadvantage complemented the components of the deprivation index. Measures of material disadvantage included educational attainment (university degree; post-secondary diploma; high school diploma; no high school diploma), income (ratio of family income to low-income cut-off [24], in quintiles) and employment status (employed; unemployed; not in labour force). Measures of social disadvantage included civil status (legally married; common-law; never married; separated/divorced/widowed), family structure (two-parent family; lone parent; non-family) and persons living alone (no; yes).

Covariates included age (25-34; 35-44; 45-54; 55-64; 65-74; 75-84; 85+ years), visible minority (no; yes - Black/Asian/Arab/Pacific Islander/Latin American/multiple), immigrant status (Canadian citizen; non-permanent resident; landed immigrant), provincial region (Atlantic; Québec; Ontario; Prairie; British Columbia) and community size (>2,500 residents; ≤2,500 residents). Covariates were chosen on the basis of their importance in relation to socioeconomic disadvantage and suicide in the literature [9,11,31].

### Statistical analysis

Hazard ratios (HR) and 95% confidence intervals (CI) were estimated with Cox proportional hazards multivariable regression for the follow-up period from 4 June 1991 (census day) to whichever came first: suicide (event), death from another cause (censored), or 31 December 2001 (censored). Time-on-study was used as the time scale. The proportional hazard assumption was verified with log(-log Survival) curves. Analyses were performed for men and women separately as previous research indicates the relationship between social and economic indicators and suicide mortality differs by sex [1,8,13,17].

Initial models were adjusted for age only, and subsequent models were fully adjusted for all covariates and area deprivation. In general, fully adjusted estimates reflect the direct (unmediated) effect of an exposure on an outcome, while the unadjusted estimates reflect the total effect (direct plus indirect) [32]. Modifying effects of area deprivation on the relation between individual-level variables and suicide were subsequently assessed with data stratified according to level of area deprivation: low (quintiles 1 and 2 combined) versus high (quintiles 4 and 5) deprivation, separately for material and social dimensions. Quintile 3 was not included in stratified analyses. Differences between HRs from high vs. low deprivation strata were assessed by computing the ratio of HRs (95% CI) using the method proposed by Altman and Bland [33]. When the confidence intervals for the ratio of HRs for deprived vs. advantaged areas exclude the null, a modifying effect of deprivation is present.

In sensitivity analyses, final models were run for suicides alone to ensure results were comparable to those for suicide plus undetermined deaths, for a follow-up period ending in 1999 to ensure the change in ICD coding in 2000 did not bias results, and using age as the underlying time [34,35]. Analyses were performed using  SAS 9.1 [36]. Clustering in EAs was accounted for using the robust sandwich estimator in select models.

## Results

There were 260,820 deaths during the study period, of which 4,000 (1.5%) were suicides. Suicide decedents tended to be older, unmarried, living alone, unemployed, less educated, low income, and living in areas with higher social and material deprivation (Table 1).

**Table 1:** Characteristics of suicide deaths,^a ^Canadian census mortality follow-up study cohort, 1991-2001

		Male		Female	
	Study population^b^ N = 2,685,400	Suicides N = 3,110	%	Suicides N = 880	%
**Individual level**					
*Age*					
25-44	1,462,800	1,790	0,25	534	0,07
45-64	809,500	910	0,21	258	0,07
65+	413,000	410	0,21	88	0,04
*Social disadvantage*					
Civil Status					
Separated/divorced/widowed	357,100	387	0,40	237	0,09
Never married	326,700	734	0,42	173	0,11
Common-law	177,900	264	0,29	67	0,08
Legally married	1,823,700	1,725	0,18	403	0,05
Family structure					
Non-family	473,500	859	0,43	272	0,10
Lone parent	169,100	172	0,38	130	0,10
Two-parent family	2,042,700	2,079	0,19	478	0,05
Persons living alone					
Yes	302,000	572	0,49	186	0,10
No	2,383,300	2,538	0,21	694	0,06
*Material disadvantage*					
Education					
No high school diploma	936,300	1,343	0,29	322	0,07
High school diploma	977,300	1,184	0,24	314	0,07
Post-secondary diploma	413,200	275	0,17	163	0,07
University degree	358,500	308	0,15	81	0,05
Income adequacy quintile					
Quintile 1 - poorest	455,900	693	0,36	298	0,11
Quintile 2	521,700	628	0,25	160	0,06
Quintile 3	556,700	624	0,22	146	0,05
Quintile 4	572,300	636	0,21	130	0,05
Quintile 5 - richest	578,800	529	0,17	146	0,05
Employment status					
Not in labour force	764,800	785	0,28	384	0,08
Unemployed	164,600	347	0,39	80	0,10
Employed	1,756,000	1,978	0,20	416	0,05
**Area level**					
*Social deprivation*					
Highest	540,000	728	0,29	287	0,10
Second highest	544,500	626	0,20	186	0,07
Middle	540,600	635	0,20	152	0,06
Second lowest	534,600	598	0,22	146	0,06
Lowest	525,700	523	0,19	109	0,04
*Material deprivation*					
Highest	531,400	832	0,31	193	0,07
Second highest	526,200	689	0,26	159	0,06
Middle	532,800	595	0,22	179	0,07
Second lowest	541,200	496	0,18	166	0,06
Lowest	553,700	498	0,19	183	0,06

**^a ^**Refers to suicide plus undetermined deaths

**^b ^**Census population counts rounded to nearest 100

### Individual-level disadvantage

All indicators of individual social and material disadvantage were associated with suicide in age-adjusted models (Table 2). Results were similar for both sexes. The risk of suicide among males and females who were separated/divorced/widowed or never married was more than twice that of legally married individuals, and the risk was also elevated for those in common-law unions. Family structure was related to suicide, particularly among men and women not living in a family relative to two-parent families. Similarly, men and women living alone had more than twice the risk of suicide relative to individuals not alone. Suicide risk decreased with greater education and income. Men and women who were unemployed or not in the labour force were at greater risk of suicide compared to their employed counterparts.

**Table 2:** Associations between suicide mortality and measures of social and material deprivation, for males and females, Canadian census mortality follow-up study cohort, 1991-2001

	Males		Females	
	**Age-adjusted HR (95% CI)**	**Fully-adjusted HR (95% CI)^a^**	**Age-adjusted HR (95% CI)**	**Fully-adjusted HR (95% CI)^a^**

**Individual level**				
*Social disadvantage*				
Civil status				
Separated/divorced/widowed	2.37 (2.12-2.65)	1.37 (1.11-1.69)	2.57 (2.17-3.05)	1.48 (0.93-2.37)
Never married	2.45 (2.23-2.68)	1.50 (1.26-1.79)	2.67 (2.23-3.21)	1.64 (1.06-2.55)
Common-law	1.64 (1.44-1.88)	1.31 (1.14-1.49)	1.62 (1.25-2.11)	1.40 (1.07-1.82)
Legally married	1	1	1	1
Overall *p *value	<0.0001	<0.0001	<0.0001	0.02
Family structure				
Non-family	2.34 (2.16-2.54)	1.21 (0.99-1.48)	2.85 (2.43-3.34)	1.56 (0.98-2.51)
Lone parent	2.00 (1.71-2.34)	1.26 (1.01-1.58)	2.11 (1.74-2.57)	1.09 (0.68-1.76)
Two-parent family	1	1	1	1
Overall *p *value	<0.0001	0.09	<0.0001	0.02
Persons living alone				
Yes	2.44 (2.22-2.67)	1.40 (1.22-1.62)	2.41 (2.02-2.87)	1.04 (0.80-1.35)
No	1	1	1	1
Overall *p *value	<0.0001	<0.0001	<0.0001	0.78
*Material disadvantage*				
Education attainment				
No high school diploma	2.10 (1.85-2.28)	1.56 (1.36-1.79)	1.58 (1.24-2.03)	1.33 (1.01-1.74)
High school diploma	1.57 (1.38-1.78)	1.36 (1.20-1.55)	1.34 (1.05-1.71)	1.32 (1.02-1.70)
Post-secondary diploma	1.08 (0.91-1.27)	1.00 (0.85-1.18)	1.32 (1.01-1.72)	1.37 (1.04-1.79)
University degree	1	1	1	1
Overall *p *value	<0.0001	<0.0001	0.003	0.13
Income adequacy quintile				
Quintile 1 - poorest	2.19 (1.95-2.45)	1.49 (1.31-1.69)	2.50 (2.04-3.05)	1.33 (1.05-1.70)
Quintile 2	1.46 (1.29-1.64)	1.25 (1.10-1.41)	1.26 (1.01-1.59)	0.91 (0.72-1.16)
Quintile 3	1.27 (1.13-1.43)	1.15 (1.02-1.30)	1.05 (0.83-1.32)	0.85 (0.67-1.08)
Quintile 4	1.23 (1.09-1.38)	1.15 (1.03-1.30)	0.90 (0.71-1.15)	0.81 (0.64-1.04)
Quintile 5 - richest	1	1	1	1
Overall *p *value	<0.0001	<0.0001	<0.0001	<0.0001
Employment status				
Not in labour force	2.18 (1.96-2.43)	1.50 (1.34-1.68)	2.24 (1.93-2.61)	2.10 (1.78-2.48)
Unemployed	1.92 (1.71-2.15)	1.48 (1.31-1.67)	2.01 (1.59-2.56)	1.91 (1.50-2.44)
Employed	1	1	1	1
Overall *p *value	<0.0001	<0.0001	<0.0001	<0.0001
**Area level**				
*Social deprivation*				
Highest	1.56 (1.39-1.75)	1.12 (0.99-1.27)	2.57 (2.06-3.21)	1.71 (1.34-2.18)
Second highest	1.25 (1.11-1.41)	1.05 (0.93-1.18)	1.68 (1.32-2.12)	1.35 (1.05-1.72)
Middle	1.23 (1.10-1.39)	1.09 (0.97-1.22)	1.38 (1.08-1.77)	1.21 (0.94-1.56)
Second lowest	1.14 (1.02-1.29)	1.07 (0.95-1.20)	1.34 (1.05-1.72)	1.27 (0.99-1.63)
Lowest	1	1	1	1
Overall *p *value	<0.0001	0.46	<0.0001	0.0003
*Material deprivation*				
Highest	1.70 (1.52-1.90)	1.02 (0.90-1.15)	1.17 (0.96-1.44)	0.83 (0.65-1.04)
Second highest	1.42 (1.27-1.60)	1.04 (0.92-1.17)	0.96 (0.78-1.19)	0.78 (0.62-0.98)
Middle	1.21 (1.08-1.37)	1.00 (0.88-1.13)	1.05 (0.86-1.30)	0.96 (0.77-1.19)
Second lowest	1.00 (0.88-1.13)	0.90 (0.79-1.02)	0.95 (0.77-1.17)	0.92 (0.74-1.14)
Lowest	1	1	1	1
Overall *p *value	<0.0001	0.16	0.26	0.19

**^a ^**Hazard ratios (95% confidence interval) adjusted for age, all variables in table, visible minority, immigrant status, provincial region of residence, and community population size

Associations were attenuated in fully adjusted models, but remained statistically significant especially for males. Women who were never married or in common-law unions, without a university degree, in the lowest income quintile, and unemployed or not in labour force remained at higher risk compared to their respective counterparts.

### Area-level deprivation

For males, suicide risk was higher in areas with high social and material deprivation in age-adjusted models, but associations were nullified in fully adjusted models (Table 2). For females, only social deprivation was associated with greater suicide risk, with associations attenuated or nullified in fully adjusted models.

### Influence of individual disadvantage according to level of area deprivation

Associations between individual disadvantage and suicide were similar when areas with low social deprivation were compared to those with high social deprivation (Tables 3 and 4). A modifying effect of social area deprivation was, however, observed in females for the influence of low income on suicide. Low income females were at higher risk of suicide relative to high income females in areas with high social deprivation, but had a lower risk of suicide relative to high income females in areas with low social deprivation.

**Table 3:** Individual measures of social and material disadvantage and risk of suicide mortality for males, according to area social deprivation, Canadian census mortality follow-up study cohort, 1991-2001

	**Low social deprivation**	**High social deprivation**	**Ratio of HRs^b^**
	**HR (95% CI)^a^**	**HR (95% CI)^a^**	**(95% CI)**
	
*Social disadvantage*			
Civil status			
Separated/divorced/widowed	1.25 (0.87-1.78)	1.48 (1.07-2.05)	0.84 (0.52-1.37)
Never married	1.56 (1.19-2.06)	1.38 (1.03-1.85)	1.13 (0.76-1.69)
Common-law	1.55 (1.23-1.94)	1.15 (0.94-1.41)	1.35 (0.99-1.83)
Legally married	1	1	
Family structure			
Non-family	1.26 (0.90-1.77)	1.10 (0.80-1.50)	1.15 (0.72-1.82)
Lone parent	1.43 (0.99-2.06)	1.11 (0.78-1.58)	1.29 (0.77-2.14)
Two-parent family	1	1	
Persons living alone			
Yes	1.63 (1.21-2.19)	1.42 (1.18-1.71)	1.15 (0.81-1.63)
No	1	1	
*Material disadvantage*			
Education attainment			
No high school diploma	1.35 (1.07-1.69)	1.72 (1.41-2.11)	0.78 (0.58-1.06)
High school diploma	1.26 (1.02-1.57)	1.40 (1.15-1.70)	0.90 (0.67-1.20)
Post-secondary diploma	0.95 (0.72-1.24)	1.04 (0.81-1.32)	0.91 (0.63-1.32)
University degree	1	1	
Income adequacy quintile			
Quintile 1 - poorest	1.34 (1.08-1.65)	1.58 (1.29-1.93)	0.85 (0.63-1.14)
Quintile 2	1.21 (0.99-1.48)	1.24 (1.02-1.51)	0.98 (0.74-1.29)
Quintile 3	1.09 (0.90-1.32)	1.15 (0.95-1.40)	0.95 (0.72-1.24)
Quintile 4	1.21 (1.02-1.44)	1.05 (0.86-1.28)	1.15 (0.89-1.50)
Quintile 5 - richest	1	1	
Employment status			
Not in labour force	1.69 (1.40-2.06)	1.44 (1.21-1.71)	1.17 (0.91-1.52)
Unemployed	1.57 (1.28-1.93)	1.47 (1.24-1.74)	1.07 (0.82-1.39)
Employed	1	1	

**^a ^**Hazard ratios (95% confidence interval) adjusted for age, visible minority, immigrant status, provincial region of residence, community population size, area material deprivation and all variables in table

**^b ^**The ratio of HRs indicates a modifying effect of area deprivation when the confidence intervals exclude 1

**Table 4:** Individual measures of social and material disadvantage and risk of suicide mortality for females, according to area social deprivation, Canadian census mortality follow-up study cohort, 1991-2001

	**Low social deprivation**	**High social deprivation**	**Ratio of HRs^b^**
	**HR (95% CI)^a^**	**HR (95% CI)^a^**	**(95% CI)**
	
*Social disadvantage*			
Civil status			
Separated/divorced/widowed	1.21 (0.53-2.77)	1.25 (0.63-2.49)	0.97 (0.33-2.84)
Never married	1.77 (0.88-3.58)	1.36 (0.70-2.65)	1.30 (0.49-3.42)
Common-law	1.21 (0.72-2.05)	1.39 (0.96-2.00)	0.87 (0.46-1.65)
Legally married	1	1	
Family structure			
Non-family	1.83 (0.83-4.04)	1.64 (0.81-3.30)	1.12 (0.39-3.21)
Lone parent	1.42 (0.62-3.26)	1.24 (0.62-2.48)	1.15 (0.39-3.38)
Two-parent family		1	
Persons living alone			
Yes	0.75 (0.37-1.50)	1.20 (0.87-1.66)	0.63 (0.29-1.35)
No	1	1	
*Material disadvantage*			
Education attainment			
No high school diploma	1.21 (0.73-2.00)	1.16 (0.81-1.65)	1.04 (0.56-1.93)
High school diploma	1.44 (0.90-2.30)	1.09 (0.78-1.52)	1.32 (0.74-2.35)
Post-secondary diploma	1.33 (0.81-2.20)	1.15 (0.81-1.64)	1.16 (0.63-2.13)
University degree	1	1	
Income adequacy quintile			
Quintile 1 - poorest	1.18 (0.79-1.79)	1.92 (1.31-2.81)	0.61 (0.35-1.08)
Quintile 2	0.61 (0.39-0.93)	1.44 (0.98-2.10)	0.42 (0.24-0.76)
Quintile 3	0.80 (0.56-1.16)	1.21 (0.82-1.78)	0.66 (0.39-1.13)
Quintile 4	0.55 (0.37-0.81)	1.13 (0.76-1.69)	0.49 (0.28-0.85)
Quintile 5 - richest	1	1	
Employment status			
Not in labour force	1.80 (1.33-2.42)	2.14 (1.69-2.71)	0.84 (0.57-1.23)
Unemployed	1.54 (0.94-2.50)	2.01 (1.45-2.78)	0.77 (0.43-1.38)
Employed	1	1	

**^a ^**Hazard ratios (95% confidence interval) adjusted for age, visible minority, immigrant status, provincial region of residence, community population size, area material deprivation and all variables in table

**^b ^**The ratio of HRs indicates a modifying effect of area deprivation when the confidence intervals exclude 1

Associations between individual disadvantage and suicide also differed little in areas with low compared to high material deprivation (Tables 5 and 6). One exception was a modifying effect of area material deprivation for males living alone. In areas with high material deprivation, males living alone were at higher risk of suicide relative to males not alone; but there was no difference in risk between these two groups in areas with low material deprivation.

**Table 5:** Individual measures of social and material disadvantage and risk of suicide mortality for males, according to area material deprivation, Canadian census mortality follow-up study cohort, 1991-2001

	**Low material deprivation**	**High material deprivation**	**Ratio of HRs^b^**
	**HR (95% CI)^a^**	**HR (95% CI)^a^**	**(95% CI)**
	
*Social disadvantage*			
Civil status			
Separated/divorced/widowed	1.46 (0.98-2.16)	1.30 (0.98-1.73)	1.12 (0.69-1.83)
Never married	1.54 (1.09-2.17)	1.43 (1.13-1.81)	1.08 (0.71-1.63)
Common-law	1.38 (1.07-1.78)	1.30 (1.08-1.56)	1.06 (0.78-1.45)
Legally married	1	1	
Family structure			
Non-family	1.45 (0.99-2.10)	1.05 (0.79-1.38)	1.38 (0.86-2.21)
Lone parent	1.25 (0.81-1.95)	1.20 (0.89-1.62)	1.04 (0.61-1.77)
Two-parent family	1	1	
Persons living alone			
Yes	1.01 (0.79-1.29)	1.78 (1.45-2.20)	0.57 (0.41-0.78)
No	1	1	
*Material disadvantage*			
Education attainment			
No high school diploma	1.54 (1.27-1.88)	1.50 (1.16-1.93)	1.03 (0.74-1.42)
High school diploma	1.27 (1.06-1.51)	1.30 (1.01-1.67)	0.98 (0.72-1.33)
Post-secondary diploma	0.88 (0.69-1.11)	1.10 (0.82-1.49)	0.80 (0.55-1.17)
University degree	1	1	
Income adequacy quintile			
Quintile 1 - poorest	1.51 (1.21-1.89)	1.43 (1.19-1.72)	1.06 (0.79-1.41)
Quintile 2	1.19 (0.97-1.47)	1.18 (0.98-1.42)	1.01 (0.76-1.33)
Quintile 3	1.31 (1.09-1.58)	1.07 (0.88-1.29)	1.22 (0.94-1.60)
Quintile 4	1.02 (0.85-1.23)	1.15 (0.96-1.39)	0.89 (0.68-1.15)
Quintile 5 - richest	1	1	
Employment status			
Not in labour force	1.41 (1.13-1.77)	1.50 (1.29-1.75)	0.94 (0.72-1.23)
Unemployed	1.49 (1.16-1.91)	1.44 (1.24-1.69)	1.03 (0.77-1.39)
Employed	1	1	

**^a ^**Hazard ratios (95% confidence interval) adjusted for age, visible minority, immigrant status, provincial region of residence, community population size, area material deprivation and all variables in table

**^b ^**The ratio of HRs indicates a modifying effect of area deprivation when the confidence intervals exclude 1

**Table 6:** Individual measures of social and material disadvantage and risk of suicide mortality for females, according to area material deprivation, Canadian census mortality follow-up study cohort, 1991-2001

	**Low material deprivation**	**High material deprivation**	**Ratio of HRs^b^**
	**HR (95% CI)^a^**	**HR (95% CI)^a^**	**(95% CI)**
	
*Social disadvantage*			
Civil status			
Separated/divorced/widowed	2.11 (0.98-4.57)	1.45 (0.75-2.81)	1.46 (0.53-4.01)
Never married	2.00 (0.98-4.10)	1.82 (1.26-2.62)	1.10 (0.49-2.45)
Common-law	1.06 (0.64-1.77)	0.97 (0.48-1.93)	1.09 (0.46-2.59)
Legally married	1	1	
Family structure			
Non-family	0.87 (0.39-1.94)	2.30 (1.14-4.65)	0.38 (0.13-1.10)
Lone parent	0.84 (0.38-1.86)	1.45 (0.72-2.93)	0.58 (0.20-1.67)
Two-parent family	1	1	
Persons living alone			
Yes	1.19 (0.76-1.86)	1.20 (0.80-1.81)	0.99 (0.54-1.82)
No	1	1	
*Material disadvantage*			
Education attainment			
No high school diploma	1.29 (0.88-1.87)	1.86 (0.99-3.50)	0.69 (0.33-1.45)
High school diploma	1.20 (0.86-1.68)	1.83 (0.97-3.43)	0.66 (0.32-1.34)
Post-secondary diploma	1.34 (0.94-1.90)	1.90 (0.98-3.69)	0.71 (0.33-1.49)
University degree	1	1	
Income adequacy quintile			
Quintile 1 - poorest	1.35 (0.94-1.95)	1.62 (1.04-2.51)	0.83 (0.47-1.48)
Quintile 2	0.95 (0.66-1.35)	1.05 (0.67-1.65)	0.90 (0.51-1.61)
Quintile 3	0.87 (0.61-1.23)	0.96 (0.61-1.51)	0.91 (0.51-1.61)
Quintile 4	0.89 (0.64-1.24)	0.93 (0.58-1.49)	0.96 (0.54-1.70)
Quintile 5 - richest	1	1	
Employment status			
Not in labour force	2.08 (1.59-2.72)	1.99 (1.53-2.59)	1.05 (0.72-1.52)
Unemployed	1.59 (1.01-2.50)	1.81 (1.26-2.61)	0.88 (0.49-1.57)
Employed	1	1	

**^a ^**Hazard ratios (95% confidence interval) adjusted for age, visible minority, immigrant status, provincial region of residence, community population size, area material deprivation and all variables in table

**^b ^**The ratio of HRs indicates a modifying effect of area deprivation when the confidence intervals exclude 1

Models excluding undetermined deaths, with follow-up ending in 1999, accounting for clustering and using age as the underlying time showed similar results (data not shown).

## Discussion

This study used a Canadian database linking detailed census data on a leading external cause of mortality, suicide [24], to investigate relations with individual disadvantage, measured both socially and materially, while accounting for social and material deprivation of residential areas. Individual social and material disadvantage were associated with suicide, particularly among males, even adjusting for area deprivation and other covariates. Suicide risk was highest among males who were never married, without a high school diploma, in the lowest income quintile, unemployed or not in the labour force. For females, suicide risk was particularly elevated for those never married or in common-law unions and those unemployed or not in the labour force. Associations for some individual-level characteristics suggested potentially greater risk of suicide in areas that were materially or socially deprived, particularly for females with low income and males living alone.

### Individual social disadvantage

Our results showed that married men and women had lower risks of suicide compared to individuals in common-law unions, or who are separated/divorced/widowed or never married. Other studies also find marriage protective, possibly because marriage confers emotional stability and reduces isolation through opportunities for social and community integration [1,8,13,14,17,31,37]. In our study, associations were of similar magnitude for males and females. This is in line with studies from Denmark, Sweden and England and Wales [13,31,38], but not US research that found non-married status increased the suicide risk only for men. US data suggest that marriage confers greater health benefits to men than women, possibly because women invest time and energy in caring for household members, and males without such social support may be prone to suicide [1]. This may be less the case in Denmark, Sweden, England and Wales, and Canada where there may be greater equality in household roles between the sexes than in the US.

Although studies report associations between civil status and suicide, few evaluate cohabitation. Two Danish studies found that cohabiting individuals were at higher risk of suicide than their legally married counterparts [13,14], supporting our results. This finding is perplexing given that common-law unions are almost equivalent to legal marital status in both Denmark and Canada. Whether the lower suicide risk associated with legal marital status is due to a protective influence of marriage (e.g., partner support during difficulties) or marriage selection (i.e., healthier individuals being more likely to marry and stay married) remains unclear [14].

Lone parenthood often implies poorer socioeconomic circumstances and health, but some studies suggest that having children may be protective against suicide in parents, especially women [11,14,39]. Adjusted associations indicated, however, no association between lone parenthood and suicide for females and a greater risk for males. In Sweden, significant associations between lone parenthood and suicide remained after adjustment for socioeconomic status for both men and women [40,41]. Compared to cohabiting custodial fathers in Sweden, lone non-custodial fathers and lone childless men were at higher suicide risk, but lone custodial fathers were not [40]. This suggests that the greater risk of suicide may have more to do with living alone than having children. We did not have access to data on custody, but the higher risk among lone fathers (possibly non-custodial) and men living alone, would correspond with the Swedish findings.

### Individual material disadvantage

We found that individual material disadvantage was associated with suicide. Material disadvantage could influence suicide risk in several ways [1]. Low levels of education may limit an individual's development of self-control or strategies for managing stressful situations, or may lead to life situations less likely to promote marriage, employment, and social capital. Our results contrast with several other studies that found that education was not associated with suicide [8-11,16]. Why education influences suicide in Canada, but not elsewhere, remains to be explored. Low education was a stronger risk factor for males than for females. The benefits of education may depend on the promise of future occupational success - a promise that may be fulfilled for men more often than women [1]. Indeed, the particularly elevated suicide risks observed for unemployed females and those not in the labour force suggest that employment, more than education, is an important predictor of suicide in women. Employment provides income, structures daily routines and offers social interaction.

### Area-level deprivation

In terms of area-level associations, our results are in line with other research [5,6] indicating greater suicide risks in areas with higher area deprivation. However, only social (and not material) deprivation was associated with suicide in females, which suggests that social characteristics of areas may be more important factors leading to suicide in women. Our finding that area-level associations were attenuated following adjustment for individual-level characteristics has been reported in some other studies [13,19] but not all [18].

### Effect modification

In line with previous European studies [13,19], stratified analyses provided little evidence that the influence of individual-level social or material disadvantage on suicide was modified by area deprivation. There was, however, some evidence that the suicide risks were elevated for low income females in socially deprived areas, but not in areas with low social deprivation. Also, the risk of suicide was greater among males living alone in areas with high material deprivation, but not in areas with low material deprivation. These results suggest that accumulation of individual and area disadvantage may increase suicide risk for some risk factors, or that well-off individuals are protected from negative influences of deprived areas. Given that some findings may be statistically significant due to chance, evidence for effect modification was not compelling.

### Limitations

We could not adjust for some covariates that could mediate or confound results, such as substance use or psychiatric illness [10,11,14], nor did we test for interaction between exposures and potential mediators [32], hence results from fully adjusted models should not be considered unbiased estimates of direct (unmediated) effects on suicide [42]. Nor could we account for time-varying covariates including civil status, family structure, living alone, place of residence, and to some extent employment status which may have changed across 10-years. Residential mobility is not likely to have influenced our results as US data suggest that people tend to move to similar places [43]. We could not account for self-selection of individuals into areas, a problem common to most area-based studies. The extent to which deprivation indices reflect true deprivation is unclear, as other unmeasured area factors such as occupation, house or car ownership could contribute [44,45]. Results may not generalize to younger adults or people in the Territories who may have different socioeconomic circumstances.

## Conclusion

Individual disadvantage was associated with suicide mortality in Canada among both sexes, particularly for individuals who were not legally married, low income or not employed. Evidence for a modifying effect of area deprivation was not substantial. Prevention strategies should primarily focus on individuals who are unemployed or out of the labour force, have low education or income. Females with low income and males who live alone in deprived areas should also be targeted.

## List of abbreviations

95% CI: (95% confidence interval); EA: (census enumeration areas); HR: (hazard ratio); ICD: (International Classification of Diseases); US: (United States).

## Conflict of interests

The authors declare that they have no competing interests.

## Authors' contributions

SB and NA conceived of the study and interpreted the results. SB reviewed the literature and drafted the article. PG and DH performed the statistical analyses. DS-L participated in the conception of the study. All authors critically reviewed the manuscript for important intellectual content, and read and approved the final version.

## Funding

Funding for the creation of the Census Mortality Follow-up Study was provided by the Canadian Population Health Initiative. SB was supported by a postdoctoral grant from the Canadian Institutes for Health Research.

## Pre-publication history

The pre-publication history for this paper can be accessed here:

http://www.biomedcentral.com/1471-2458/11/577/prepub
